# Lipid and Protein Co-Regulation of PI3K Effectors Akt and Itk in Lymphocytes

**DOI:** 10.3389/fimmu.2015.00117

**Published:** 2015-03-13

**Authors:** Xinxin Wang, Leonard Benjamin Hills, Yina Hsing Huang

**Affiliations:** ^1^California Institute for Biomedical Research, La Jolla, CA, USA; ^2^Department of Microbiology and Immunology, Geisel School of Medicine at Dartmouth, Lebanon, NH, USA; ^3^Department of Pathology, Geisel School of Medicine at Dartmouth, Lebanon, NH, USA

**Keywords:** PI3K, lymphocyte activation, pleckstrin homology domain, Akt signaling, Itk signaling

## Abstract

The phosphoinositide 3-kinase (PI 3-kinase, PI3K) pathway transduces signals critical for lymphocyte function. PI3K generates the phospholipid PIP_3_ at the plasma membrane to recruit proteins that contain pleckstrin homology (PH) domains – a conserved domain found in hundreds of mammalian proteins. PH domain–PIP_3_ interactions allow for rapid signal propagation and confer a spatial component to these signals. The kinases Akt and Itk are key PI3K effectors that bind PIP_3_ via their PH domains and mediate vital processes – such as survival, activation, and differentiation – in lymphocytes. Here, we review the roles and regulation of PI3K signaling in lymphocytes with a specific emphasis on Akt and Itk. We also discuss these and other PH domain-containing proteins as they relate more broadly to immune cell function. Finally, we highlight the emerging view of PH domains as multifunctional protein domains that often bind both lipid and protein substrates to exert their effects.

## Lymphocyte Activation Receptors Signal through Class I PI3Ks

**Figure 1:**
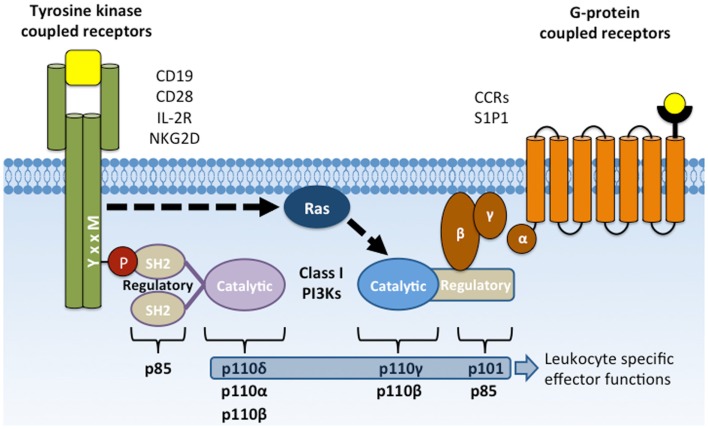
**Activation of class I PI3Ks by YxxM signaling subunits and GPCRs**. Membrane receptors that activate PI3K include CD19, CD28, and NKG2D co-receptors, cytokine receptors (e.g., IL-2R), G-protein-coupled receptors (chemokine receptors), and Fcγ receptor I and III. Class IA PI3Ks are recruited to the plasma membrane through SH2 domain interactions with phosphorylated YxxM motifs. Class IB PI3Ks are recruited and activated by direct interaction with the Gβγ subunit following GPCR activation. Activated PI3K phosphorylates the membrane lipid PI(4,5)P_2_ to form PI(3,4,5)P_3_.

Phosphoinositide 3-kinase (PI3K) activation is important for lymphocyte survival, activation, differentiation, and migration. Many lymphocyte surface receptors activate class 1 PI3Ks, which phosphorylate phosphatidyl inositol 4,5-bisphosphate [PI(4,5)P_2_, PIP_2_] at the D-3 hydroxyl group of the myo-inositol ring to generate phosphatidyl inositol 3,4,5-trisphosphate [PI(3,4,5)P_3_, PIP_3_]. Two subclasses, 1A and 1B, are activated by distinct receptor types (Figure 1). Receptors or signaling adapters that are phosphorylated at YxxM sequence motifs signal though class IA PI3K, which includes p85α and p85β regulatory subunits and p110α, p110β, and p110δ catalytic subunits. These receptors include CD19, CD28, and ICOS co-receptors; IL-2, IL-7, IL-3, IL-15, and GM-CSF cytokine receptors (1–6); and receptors coupled to TRIM, DAP10, and MyD88 adapter proteins (7–11). Receptor ligation leads to tyrosine phosphorylation at the YxxM motif and subsequent recruitment of PI3K regulatory subunits through one or both Src homology 2 (SH2) domains. Regulatory subunits are then phosphorylated by Syk or Jak family tyrosine kinases to trigger activation of their constitutively associated catalytic subunits (3).

G-protein-coupled receptors (GPCRs) signal through Class 1B PI3K, which includes p101 regulatory and p110γ catalytic subunits (12). These classic, seven transmembrane domain receptors include chemokine receptors and signal through heterotrimeric G proteins, Gα and Gβγ to promote cell migration. GPCR ligation dissociates the Gβγ dimer, allowing its binding to p101 regulatory subunits and subsequent activation of associated p110γ catalytic subunits. Activation of p110γ catalytic activity can also be induced by Ras activation (Ras-GTP) to promote migration of neutrophils (13).

Although many receptors activate class 1 PI3K, the magnitude and kinetics of PI3K activation differs greatly among receptors, depending on ligand binding kinetics and feedback circuitry that can either amplify or dampen PI3K signaling (14). Additionally, co-ligation of receptors, such as the T cell receptor (TCR) and the CD28 co-receptor, can cooperate to potentiate and sustain PI3K activation and PIP_3_ generation.

## PIP_3_ Association with Pleckstrin Homology Domains

**Figure 2:**
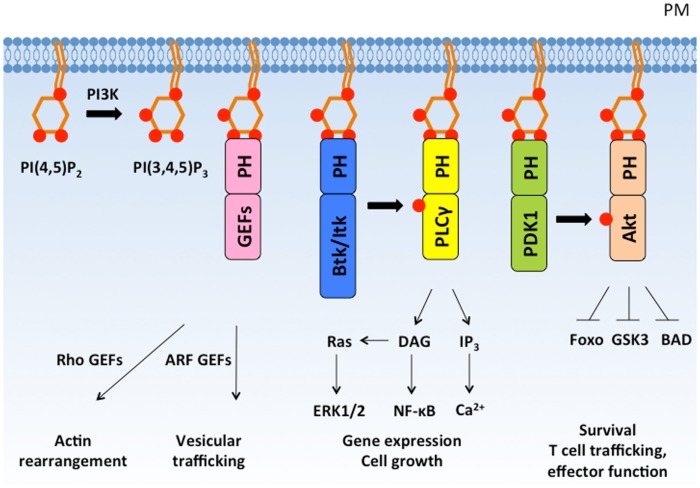
**PI(3,4,5)P_3_ recruits PH domain-containing proteins to the plasma membrane and regulates diverse cellular responses**. PI3K phosphorylates PI(4,5)P_2_ to form PI(3,4,5)P_3_, which recruits PH domain-containing signaling proteins to the plasma membrane. PH domain-containing proteins are activated at the plasma membrane and mediate important cellular responses such as cytoskeleton rearrangement, cell growth, proliferation, and survival. PM, plasma membrane; GEF, guanine nucleotide exchange factor.

PI3K activation induces PIP_3_ accumulation, which comprises less than 5% of PIP_2_ levels and less than 1% of total membrane lipids (15). Despite its low overall abundance, super-resolution microscopy has revealed ~100 nm membrane clusters of PIP_3_ that create high local PIP_3_ concentrations (16). High affinity and specificity binding between PIP_3_ and pleckstrin homology (PH) domains of PI3K effectors helps to recruit and activate these effectors at the plasma membrane (Figure 2). Like protein–protein interactions that are induced by phosphorylation, PIP_3_ interactions with PH domains allow rapid transduction of downstream signals without new protein synthesis.

**Figure 3:**
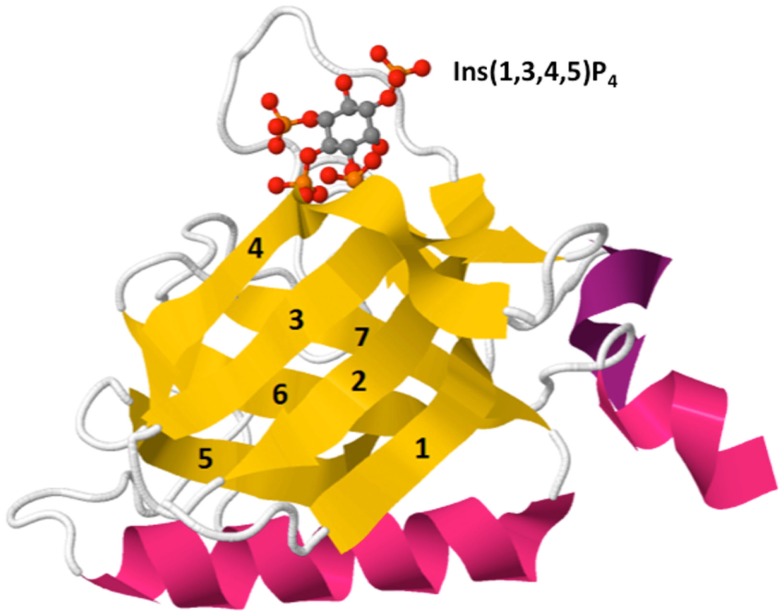
**Crystal structure of Btk PH domain in complex with Ins(1,3,4,5)P_4_ (PDB ID: 2Z0P)**. The PH domain is comprised of a β-barrel formed by seven β-strands (yellow, 1–7) capped by an α-helix (pink). The hyper-variable loops of the β-barrel form the binding surface for lipid ligands such as Ins(1,3,4,5)P_4_ [top, shown by ball-and-stick model: red (oxygen), orange (phosphorus), and gray (carbon)].

The PH domain is an evolutionarily conserved structural fold found in proteins expressed in organisms ranging from yeast to mammals (17). The core of the PH domain is a seven-strand β-barrel that is encoded by approximately 120 amino acids and is composed of two anti-parallel β sheets and a C-terminal α helix (Figure 3). The mammalian genome contains roughly 300 PH domains found in proteins that perform diverse functions including cellular activation, cytoskeletal reorganization, vesicular trafficking, and cell cycle control. Approximately, 15% of PH domains, including Akt and Itk, bind to phosphoinositides with high specificity and affinity (*K*_d_: nanomolar – low micromolar range). PH domains generally interact with phosphoinositides through positively charged lysine and arginine residues within the basic motif KXn(K/R)XR (18). However, not all PH domains bind to PIP_3_. Several PH domains interact with phosphoinositides that are selectively enriched in other membrane compartments, such as PI4P within the Golgi membrane (19) or PIP_2_ at the resting plasma membrane (17). Thus, conveying lipid specificity to PH domains constitutes a key mechanism for spatially sequestering distinct effector proteins within cells. Regulating the abundance of lipids either in resting or activated cells controls basal and induced effector activity. Additionally, regulated production of lipid ligands such as PIP_3_ within specific membrane nano-domains can induce polarized activation of downstream effectors in a robust but transient manner. This is because PIP_3_ abundance is not only spatial restricted but also finely controlled by receptor-induced PI3K-dependent PIP_3_ generation and by phosphatase and tensin homolog deleted on chromosome 10 (PTEN) and SH2 domain-containing inositol 5′-phosphatase (SHIP) phosphatase-dependent PIP_3_ metabolism.

## Protein Phosphatases Inhibit PI3K Activation While Inositol Phosphatases Reduce PIP_3_ Levels

PI3K signaling is negatively regulated at distinct steps in its signaling cascade by both protein and lipid phosphatases. Protein tyrosine phosphatases SHP-1 and SHP-2 inhibit PI3K activation by preventing early receptor signaling and by directly down-regulating PI3K activity, the latter of which is accomplished by de-phosphorylation of phospho-tyrosine residues within signal adapter proteins and PI3K regulatory subunits (71). Inhibitory receptors that restrict lymphocyte activation through SHP-1 or SHP-2 include inhibitory killer-cell immunoglobulin-like receptors (KIR) on NK cells (72), CD22 on B cells (73), and CTLA-4 and PD-1 on T cells (74, 75). Phosphorylated immunoreceptor tyrosine-based inhibition motifs (ITIM) within the cytoplasmic domains of KIRs, CD22, and CTLA-4 recruit SHP-1 and SHP-2 to prevent activating signals at the plasma membrane (72, 74, 75). Persistent T cell activation signals can also be inhibited by SHP-1 and SHP-2 recruitment to the immunoreceptor tyrosine-based switch motif (ITSM) in PD-1, an inhibitory receptor expressed on chronically stimulated T cells (76, 77). For a detailed discussion regarding the requirements of SHP-1 and SHP-2 in T cell development, differentiation, and effector function, refer to Ref. (78).

In T cells, CTLA-4 can also directly repress Akt signaling by recruiting the Ser/Thr phosphatase PP2A (77), which dephosphorylates the T308 (79, 80) and possibly S473 (79), residues required for Akt activity. Thus, CTLA-4 utilizes a dual approach to antagonize CD28 and PI3K signaling: SHP-2-dependent inhibition of TCR signaling by CD3ε de-phosphorylation and PP2A-dependent de-phosphorylation of Akt (74, 77, 81).

Lipid and inositol phosphatases also prevent PI3K effector activation. PTEN and SHIP both dephosphorylate membrane PIP_3_. However, while PTEN converts PIP_3_ back to its lipid precursor PI(4,5)P_2_ to prevent further activation of PI3K effectors, SHIP converts PIP_3_ into PI(3,4)P_2_, a lipid that retains the ability to bind the Akt PH domain (82). In the latter case, subsequent de-phosphorylation of PI(3,4)P_2_ into PI(3)P by the inositol phosphatase, INPP4B is required to “turn off” Akt membrane recruitment (83). Inhibitory receptors including FcγIIB on B cells and mast cells and Ly49A and Ly49C on NK cells contain ITIM motifs that recruit SHIP through its SH2 domain (84, 85). Membrane receptors with cytosolic PDZ domains recruit PTEN to control PIP_3_ levels. Although the functional significance of PDZ domain-containing receptors on lymphocyte activation requires additional investigation, maintaining appropriate PTEN levels is crucial for the control of immune cell homeostasis and function (86).

## General and Cell Type-Specific Akt Functions

Akt belongs to the AGC family of Serine/Threonine kinases. The three Akt isoforms are differentially expressed in various cell types but are 77–83% sequence identical. Akt activity prevents apoptosis, promotes protein expression, and regulates cellular metabolism (20–23). Akt mediates these general cellular functions through direct phosphorylation of RxRxxS*/T* motifs (24) found in a plethora of cellular targets including forkhead box transcription factors, TSC2, GSK3, and BAD, which are discussed in detail elsewhere (20). A somatic mutation in Akt that replaces glutamate with lysine at residue 17 (hereafter referred to as E17K) leads to cellular transformation and has been identified in human breast, colorectal, and ovarian cancer (25, 26). The E17K mutation is located in the lipid binding pocket of Akt’s PH domain and dramatically increases its affinity for membrane lipids, causing constitutive Akt signaling (27). Ectopic expression of E17K in hematopoietic stem cells is sufficient to induce development of lymphoblastic T cell lymphoma within 6–8 weeks following transfer into recipient mice (28). Similarly, conditional deletion of the Akt targets Foxo1/3/4 in mice leads to development of the same type of lymphomas 15–25 weeks after induction of Foxo deletion (29).

In lymphocytes, Foxo proteins regulate the gene expression of Rag recombinases, Ikaros, CCR7, IL-7R, TCF7, Eomes, and Foxp3, which are critical for controlling lymphocyte development, trafficking, and differentiation (30–37). Akt phosphorylation of Foxo1 and Foxo3 leads to their degradation and down-regulates Foxo-dependent gene expression (31, 38). Genetic ablation of both Foxo1 and Foxo3 causes a multi-focal autoimmune disease due to defective Foxp3 expression and T regulatory (Treg) cell specification and function (34). Similarly, retroviral expression of constitutively active myristoylated Akt (myrAkt) in CD4^−^CD8^−^ thymocytes impairs Treg development *in vivo* following intrathymic transfer. Importantly, the inhibitory effect of myrAkt is on *de novo* but not established Foxp3 expression (39). In contrast, broad expression of myrAkt as a transgene under the control of the CD2 promoter leads to increased regulatory T cell numbers *in vivo* and enhanced suppressive activity (40). Interestingly, conventional CD4^+^ T cells expressing transgenic myrAkt are less responsive to TGFβ suppression and fail to differentiate into the Th17 lineage in response to TGFβ and IL-6 *in vitro* (40).

A proper balance of Akt activity is also required for appropriate CD8^+^ T cell maturation, effector function, and memory development (41). Uzel and colleagues recently published a study on patients with somatic dominant active p110δ (a catalytic subunit of PI3K) expression (42). T cell blasts from these patients have increased phosphorylation of AKT at T308 and S473, a decline in Foxo1, increased S6 activation, and glucose uptake. This hyperactive Akt/mTORC1 axis causes CD8 T cells to proliferate more vigorously, differentiate more readily into effector cells, and undergo cellular senescence. Sustained Akt activity in these patients also impairs development of CD8 memory T cells, which require a metabolic “switch” from glycolysis to fatty acid oxidation (41, 43). Furthermore, defective CD8 responses result in recurrent sinopulmonary infections and chronic viremia due to Epstein-Barr virus (EBV) and/or cytomegalovirus (CMV) infection (42). Cantrell and coworkers published a surprising finding demonstrating distinct roles for PDK1 and Akt in promoting cellular metabolism and effector responses of CD8 T cells, respectively (44). T cells expressing a catalytically inactive p110δ or treated with an Akt inhibitor are defective for Akt T308 phosphorylation. Akt-defective CD8 T cells proliferate normally in response to IL-2 but are unable to express proper lymphoid homing receptors and cytotoxic effector proteins (44). In contrast, conditional deletion of PDK1, the upstream activator of Akt, leads to defective glucose uptake and metabolism, resulting in reduced CD8 T cell proliferation. This indicates that PDK1 promotes proliferation in an Akt-independent manner (44). It remains to be determined whether PDK1 and Akt have distinct roles in cell types in which multiple functions have been attributed to Akt activity.

## Tec Family Kinases Regulate Immune Cell Development and Function

The Tec family of non-receptor tyrosine kinases, including Tec, Btk, Itk/Emt/Tsk, Rlk/Txk, and Bmx/Etk, are differentially expressed in immune cells. Each Tec family member contains an N-terminal PIP_3_-binding PH domain except Rlk, which contains a cysteine-string motif that results in Rlk palmitoylation. In general, Tec kinases activate PLCγ to trigger Ca^2+^ and diacylglycerol (DAG) signaling. Mimicking Ca^2+^ and DAG activation with the addition of calcium ionophores and phorbol myristate acetate (PMA) is sufficient to induce many aspects of lymphocyte activation, differentiation, and effector responses *in vitro*. The requirement for Tec kinases in immune functions is apparent from the profound defects observed in human patients carrying mutations in Tec kinases and in mouse models of single and combined Tec kinase deficiencies.

In 1993, Btk was first identified in patients with X-linked agammaglobulinemia (XLA), an inherited immunodeficiency disease characterized by profound hypogammaglobulinemia due to severely decreased B cell numbers (45). XLA patients carry Btk mutations that prevent the maturation of pro-B cells into pre-B cells. Pre-B-cell receptor signaling at the pro-B to pre-B transition requires Btk activation by the Src kinase Lyn (46–48). A Btk mutation database generated from approximately 400 XLA patients indicates that the majority of missense mutations in the Btk PH domain are in the putative PIP_3_-binding pocket (49–51). The XLA missense mutants F25S, R28H, T33P, V64F, and V113D dramatically reduce Btk binding to PIP_3_ *in vitro* and disrupt Btk activation in B cells (52, 53). A similar mutation in mice, R28C also abolishes Btk binding to PIP_3_ and results in murine X-linked immunodeficiency (Xid) disease (53). These findings demonstrate the importance of PI3K-dependent PIP_3_ generation for the membrane recruitment and activation of Btk in promoting B cell receptor signaling during maturation and humoral immune responses.

While disruption of PIP_3_ association causes hypo-B-cell responses, enhanced PIP_3_ association also leads to B cell dysfunction. The Btk E41K mutant significantly increases Btk PH domain affinity for phosphoinositides and results in constitutive membrane localization when expressed ectopically in COS-7 cells (52, 53). Btk E41K expression allows cytokine-independent growth of the pro-B-cell line Y16 (54), demonstrating its gain-of-function activity. However, mice expressing a Btk E41K transgene controlled by the MHC class II locus are more severely B cell-deficient than even Xid mice (55). Lack of IgM^high^ cells in the bone marrow suggest that constitutive Btk E41K activation leads to inappropriate deletion of immature B cells by mimicking strong BCR signals that promote apoptosis of auto-reactive B cells (55). Thus, appropriate levels of Btk activation are critical for developmental progression of B cells, productive B cell activation and differentiation, as well as deletion of auto-reactive cells.

The first patients identified with Itk mutations were initially diagnosed with Hodgkin’s lymphoma but subsequently characterized to have an underlying immunodeficiency disease that prevents control of EBV-induced B cell proliferation (56). Itk-deficient patients have decreased T cells (57), which are required to control EBV infection and prevent viral reactivation from latently infected B cells (58). Detailed characterization of Itk-deficient mice reveals multiple requirements for Itk during T cell development, differentiation, and function (59, 60). Like Btk in B cells, Itk participates in proximal antigen receptor signaling and is directly phosphorylated by a Src family kinase, in this case Lck (61). Activated Itk phosphorylates PLCγ1, which induces IP_3_-dependent increased intracellular Ca^2+^ levels as well as DAG-mediated signaling (59, 62, 63). Itk is required for efficient CD4^+^ T cell differentiation toward the Th2 and Th17 lineages (59). Itk-deficient mice cannot generate protective Th2 responses in multiple infection models, including *Leishmania major*, *Nippostrongylus brasiliensis*, and *Schistosoma mansoni* (59, 64). Defective Th2 differentiation is accompanied by substantially reduced production of the Th2 cytokines IL-4, IL-5, and IL-13 by Itk-deficient T cells (65, 66). Itk is also required for optimal production of the Th17 cytokine, IL-17A but not IL-17F (67). The selective requirement for Itk in IL-17A production is mechanistically linked to a requirement for the transcription factor nuclear factor of activated T cells (NFAT) in IL-17A transcription (64, 67, 68). Prolonged Itk activation maintains cytosolic Ca^2+^ levels to promote sustained calcineurin-dependent NFAT nuclear translocation. Itk deficiency or suboptimal TCR signaling restricts autoimmunity by biasing T cell differentiation from the Th17 toward the regulatory T cell lineage (69). In addition, autoimmune organ destruction can be limited by Itk-dependent control of transendothelial migration and tissue infiltration of effector T cells (70). Thus, mechanisms that regulate the magnitude and kinetics of Itk activity in T cells are important for induction of effector functions, specification of appropriate T cell lineages, and control of T cell trafficking.

## Soluble Analogs of PIP_3_ Differentially Regulate PIP_3_ Effectors

**Figure 4:**
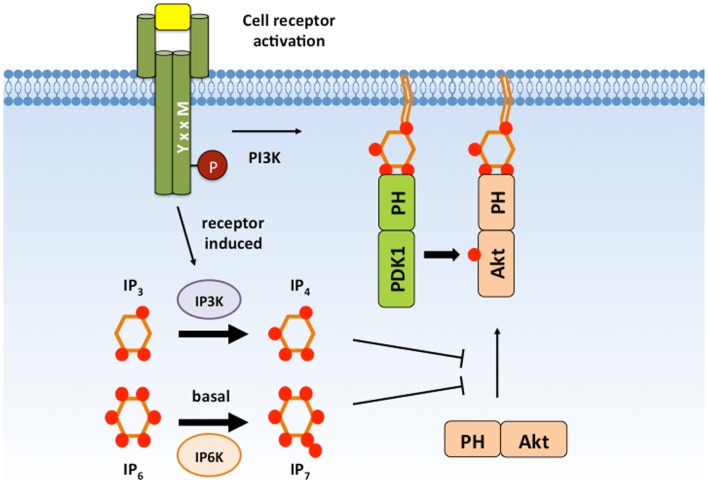
**IP_4_ and IP_7_ negatively regulate Akt signaling**. IP_4_ and IP_7_ are cytosolic PIP_3_ analogs that are able to associate with the Akt PH domain with high affinity and can compete with membrane PIP_3_. IP_4_ and IP_7_ binding has been proposed to dissociate Akt from the plasma membrane to prevent Akt activation and substrate accessibility. IP_4_, Ins(1,3,4,5)P_4_; IP_7_, 5-PP-(1,2,3,4,6)IP_5_; PIP_3_, PI(3,4,5)P_3_.

Some PIP_3_-binding PH domains can associate with soluble PIP_3_ analogs. These include the cytosolic inositol phosphates Ins(1,3,4,5)P_4_ (IP_4_), Ins(1,2,3,4,5,6)P_6_ (IP_6_), and 5-PP-I(1,2,3,4,6)P_5_ (IP_7_) that are generated inducibly or constitutively by distinct inositol kinases (82). The effect of IP_4_, IP_6_, and IP_7_ binding is distinct for different PH domains and cell types (Figure 4).

The inositol kinases IP_3_ kinase (Itpk) isoforms A, B, and C, and inositol polyphosphate multikinase (IPMK) can each generate IP_4_ by phosphorylating Ins(1,4,5)P_3_ (IP_3_) at the D-3 hydroxyl group [reviewed in Ref. (87)]. However, mice deficient in the ubiquitously expressed ItpkC or IPMK isoforms or in the neuronally enriched ItpkA isoform have no detectable immune abnormalities. In contrast, ItpkB expression is selectively enriched in hematopoietic cells and catalytically activated by the Ca^2+^-sensing protein calmodulin (CaM) following antigen receptor signaling. Analysis of ItpkB-deficient mice revealed a non-redundant requirement for ItpkB in lymphocyte development and activation (88–92). ItpkB deficiency results in severely reduced peripheral T cell numbers due to an absolute block in positive selection of CD4^+^CD8^+^ thymocytes (88). Defective activation of the Ras/MAP kinase pathway contributes to the T cell developmental defect (88, 89, 93). However, ItpkB-deficient CD4^+^CD8^+^ thymocytes are also defective in activation of Itk and its downstream effector PLC γ1 in response to TCR engagement (93). Itk fails to localize to the plasma membrane or assemble with the adapter protein LAT in the TCR signalosome of ItpkB-deficient thymocytes, indicating a requirement for IP_4_ in promoting Itk interactions (93). Interestingly, addition of IP_4_ increases binding of recombinant Itk PH domain to PIP_3_-coated beads *in vitro*, suggesting that IP_4_ may alter Itk PH domain conformation to enhance PIP_3_ accessibility (93).

Distinct from its effect on Itk, IP_4_ suppresses Akt activity by directly competing with PIP_3_ for binding to the Akt PH domain (94). ItpkB-deficient mice develop profound alterations in neutrophil and NK cell functions due to enhanced Akt activity during their development and activation (94, 95). Addition of membrane permeable IP_4_, but not an isomer, to the myeloid cell line HL-60 disrupts membrane localization of an Akt PH domain fused to GFP (94). In ItpkB-deficient neutrophils, Akt phosphorylation is enhanced in response to the bacterial peptide Formyl–Methionyl–Leucyl–Phenylalanine (fMLP). Enhanced Akt signaling in ItpkB-deficient neutrophils contributes to augmented anti-microbial and chemotaxis responses (94). The magnitude and kinetics of Akt phosphorylation are also increased in ItpkB-deficient NK cells (95). Elevated IFNγ secretion, granule exocytosis, and tumor cell lysis by ItpkB-deficient NK cells can be suppressed by Akt inhibition (95). Together, these studies indicate that IP_4_ dampens Akt activity in neutrophils and NK cells to restrict effector functions. Whether this occurs to shut-off innate functions during the resolution phase of an immune response or as a check to limit inflammatory damage remains unclear.

Similar to IP_4_, IP_7_ also competes with PIP_3_ for binding to the Akt PH domain and negatively regulates its activity (96). IP_7_ is generated by pyro-phosphorylation of IP_6_ at the 5-phosphate group by IP_6_ family kinases, IP6Ks (97, 98). While the importance of IP6K1 in lymphocyte function remains to be determined, analysis of IP6K1-deficient neutrophils demonstrates similar functional defects as ItpkB-deficient neutrophils. Both deficiencies result in enhanced fMLP-induced chemotaxis, superoxide production, and bacterial killing (94, 99). Akt membrane localization and activation are significantly increased in IP6K1-deficient neutrophils (99). Interestingly, IP_7_ is readily detectable in resting HL-60 cells but rapidly decreases upon fMLP stimulation (99). This suggests that IP_7_ may act to suppress initial Akt activation while IP_4_ regulates subsequent Akt activity following its induced production. Precise regulation of basal and induced IP_4_ and IP_7_ levels may act together to control the magnitude and kinetics of Akt activation in these innate immune cells. Future studies are required to determine the functional effects of IP_4_ and IP_7_ on Akt-dependent regulation of lymphocyte differentiation and effector responses. It also remains to be determined whether IP_7_ acts on other PIP_3_ effectors in immune cells as it does in *Dictyostelium discoideum* (100) or whether selective IP_7_ binding allows regulation of a particular subset of PIP_3_ effectors.

Recently, biochemical and structural analyses of Btk identified a new activating function for the inositol phosphate, IP_6_ (101). As with PIP_3_-containing liposomes, addition of soluble IP_6_ induces Btk trans-phosphorylation and activation. However, IP_6_ promotes Btk activation by an unconventional mechanism that is independent of the PIP_3_-binding pocket and membrane recruitment. Analysis of the co-crystal structure of IP_6_ with the Btk PH domain reveals an additional peripheral IP_6_ binding site sandwiched between two PH modules, termed the Saraste dimer. Molecular dynamics simulations suggest that IP_6_ neutralizes electrostatic forces in the monomer that oppose dimer formation. Mutation of the IP_6_ peripheral binding site disrupts transient dimerization and significantly abrogates IP_6_-dependent Btk trans-phosphorylation (101). IP_6_-induced Btk activation in solution represents a new PI3K-independent mechanism for controlling Btk activity. Considering that IP_6_ levels are basally high in lymphocytes, it will be important in future studies to determine whether IP_6_ contributes to tonic or B cell receptor-induced Btk function.

## Proteins Interact with and Regulate the Activity of PH Domain-Containing Proteins

Although the Akt and Itk PH domains specifically bind to PIP_3_ with (nanomolar) affinities, only ~40 mammalian PH domains appear to be PIP_3_-regulated according to Teruel and colleagues, who developed a prediction algorithm based on experimental analyses of 130 mouse PH domains (102). The majority of PH domains do not interact with lipids or bind lipids promiscuously or with low affinity (*K*_d_ ≥ 10 μM). Furthermore, a growing number of PH domains have been reported to participate in inter- and/or intra-molecular protein interactions (discussed below). These findings support a revised view of PH domains as diverse, multifunctional domains that bind lipids, proteins, or both to regulate the activity of their parent proteins.

T and B cells induce Ca^2+^ and DAG-mediated signaling through PLCγ1- and PLCγ2-mediated cleavage of PIP_2_ (103, 104). T cell-specific ablation of PLCγ1 causes defects in thymocyte selection during T cell development, reduced T cell proliferation and cytokine secretion, and the development of autoimmunity resulting from defective regulatory T cells (104). PLCγ2 plays important roles in regulating B cells, neutrophils, mast cells, and dendritic cells (105–107). PLCγ1 and PLCγ2 both contain two PH domains. The conventional, N-terminal PH domain associates with PIP_3_ (108); however, the C-terminal PH domain is interrupted by an intervening amino acid sequence comprising two tandem SH2 domains and an SH3 domain (109, 110). This split PH domain is also critical for substrate binding (111). The C-terminal half of the PLCγ1 split PH domain associates with a partial PH domain in TRPC3 (112, 113), a Ca^2+^ channel that can mediate Ca^2+^ entry in T cells. The formation of this inter-molecular PH-like domain allows PLCγ1 to bind to its substrate PIP_2_ and is critical for TRPC3 membrane targeting and surface expression (113). Conversely, the split PH domain of PLCγ2 interacts with the small GTPase Rac2, which mediates PLCγ2 activation and localization to the plasma membrane (114–116).

Pleckstrin homology domains also participate in intra-molecular interactions. In resting cells, the Akt PH domain associates with the kinase domain (KD) to maintain a closed conformation in which the activation loop is blocked (117, 118). PIP_3_ binding to the Akt PH domain exposes the activation loop, allowing T308 and S473 to be accessed and phosphorylated by PDK1 and mTORC2, respectively (119). Phosphorylation of T308 and S473 fully activates Akt and keeps the activation loop “open” for substrate docking (117–119). PH domain mutations that disrupt PH–KD interaction (e.g., L52R and Q79K) result in constitutive Akt activation (119).

**Figure 5:**
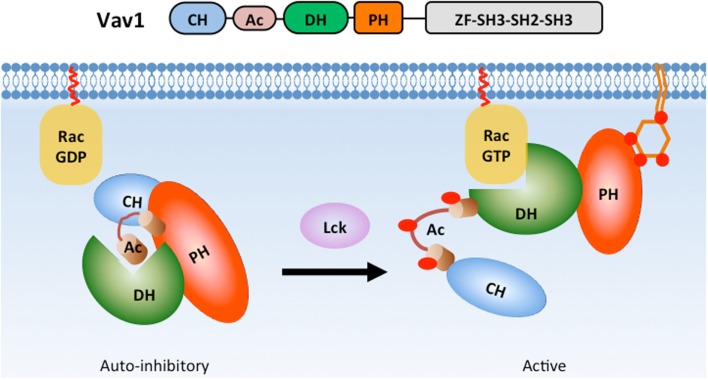
**PH domain interactions stabilize Vav1 auto-inhibition in basal state**. In the basal state, Vav1 adopts an auto-inhibitory conformation in which the substrate-docking site within the DH domain is blocked by interactions with a helix region from the Ac domain. The interactions between CH, PH, and Ac domains greatly strengthen the auto-inhibitory conformation (left). During T cell activation, phosphorylation of the Ac domain by Lck releases the substrate-docking site and allows GTPase binding (right).

The Dbl family RhoGEF Vav is also regulated by lipid and intra-molecular interactions involving its PH domain (Figure 5). Vav plays crucial roles during T cell and B cell development (120, 121) and T cell, B cell, neutrophil, and NK cell activation (9, 107, 120–123). Vav contains a Dbl homology (DH) domain that promotes the activation of the small GTPase Rac in response to PI3K activation (124, 125). In quiescent cells, Vav1 adopts an auto-inhibitory conformation, which is stabilized by interactions between its PH, acidic (Ac), and calponin homology (CH) domains (126, 127). A truncation mutation of the Vav N-terminal CH domain was shown to have oncogenic potential (128), highlighting the importance of these intra-molecular interactions in limiting Vav activity. During T cell activation, Lck phosphorylates tyrosine residues within the Ac domain to release Vav1 from auto-inhibition (127). PIP_3_ binding to the PH domain significantly enhances Lck-dependent Vav1 phosphorylation *in vitro* (129) and promotes GEF activity (124, 129, 130) likely through the release of auto-inhibition (131). Interestingly, PIP_2_ binding to the Vav1 PH domain inhibits GEF activity (129). Thus, distinct lipids bind to the Vav1 PH domain to promote conformational changes that either reinforce or relieve its auto-inhibitory state.

**Figure 6:**
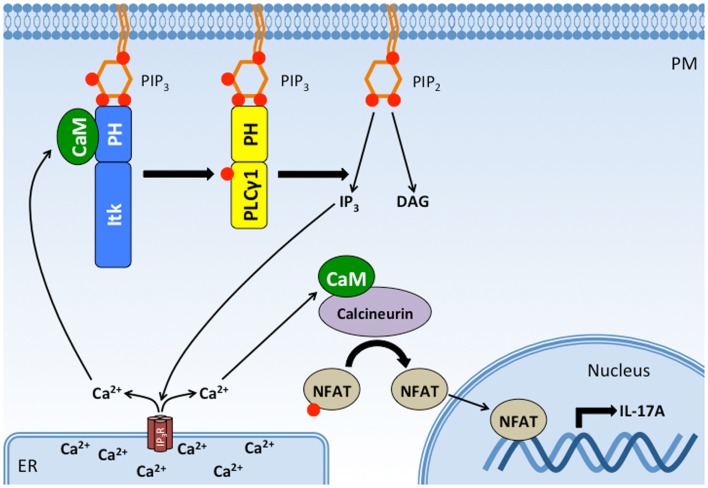
**CaM binds the Itk PH domain in a positive feedback loop that potentiates Itk activity, intracellular Ca^2+^ release, and IL-17A production**. Binding of Itk to PIP_3_ promotes Itk activation and the subsequent phosphorylation and activation of PLC γ1. PLCγ1 cleaves PIP_2_ to produce DAG and IP_3_, which binds IP_3_ receptors on the ER. The IP_3_ receptor is a ligand-gated Ca^2+^ channel, and its activation increases Ca^2+^ levels in the cytosol. Increased cytosolic Ca^2+^ activates CaM, which has at least two effects on T cell activation: (1) Ca^2+^/CaM binds to Itk’s PH domain, enhancing its interaction with PIP_3_ and Itk activity. (2) Ca^2+^/CaM binds to and activates calcineurin, a phophatase that dephosphorylates NFAT, allowing NFAT translocation to the nucleus where it drives the transcription of IL-17A. Thus, CaM binding to Itk’s PH domain completes a positive feedback loop that potentiates the downstream effects of Itk. PM, plasma membrane; ER, endoplasmic reticulum; Itk, IL-2-inducible tyrosine/T cell kinase; PLCγ1, phospholipase C gamma 1; CaM, calmodulin; NFAT, nuclear factor of activated T cells; IP_3_R, IP_3_ receptor.

Pleckstrin homology domains can also participate in inter-molecular interactions with other proteins. The PH domain of Dbs, a Cdc42/RhoGEF, associates with Cdc42 through the β3/β4 loop of its PH domain to improve substrate docking and catalysis (132). Interestingly, we recently identified the β3/β4 loop of the Itk PH domain as an important binding site for the ubiquitous Ca^2+^-sensing protein CaM (133). The CaM C-terminal EF hands bind to the β3/β4 loop of the Itk PH domain at basal intracellular Ca^2+^ levels while the CaM N-terminal EF hands engage the β5/β6 loop upon an increase in Ca^2+^ levels. CaM and PIP_3_ (but not IP_4_) reciprocally enhance binding of one another to the Itk PH domain *in vitro*, suggesting that CaM and PIP_3_ cooperate to regulate Itk signaling at the plasma membrane. Pharmacological inhibition of Ca^2+^/CaM activity or mutation of the CaM-binding β3/β4 loop disrupts Itk-dependent activation of PLCγ1 and downstream Ca^2+^ responses (133), indicating that CaM participates in a positive feedback loop whereby binding of CaM to the Itk PH domain enhances further Itk activation and downstream Ca^2+^ responses. Importantly, this positive feedback is required for optimal TCR-induced, NFAT-dependent production of the pro-inflammatory cytokine, IL-17A (133). Thus, CaM represents a novel protein-binding partner for the Itk PH domain that serves an important function in potentiating T cell pro-inflammatory responses (Figure 6). It remains to be determined how CaM, PIP_3_, and IP_4_ coordinate to regulate the kinetics and magnitude of Itk activation and whether they differentially participate in Itk-dependent T cell activation, differentiation, and effector responses.

Calmodulin has also been reported by Dong and colleagues to bind the PH domain of Akt family kinases (134). Using short peptide fragments of Akt1 in a pulldown assay, this interaction was further mapped to the first 42 residues of the Akt1 PH domain. Although CaM did not directly alter Akt kinase activity, CaM was reported to reduce the ability of PIP_3_ to co-precipitate Akt (134), suggesting that CaM competes with PIP_3_ to dampen Akt activity. However, this finding is inconsistent with other published data demonstrating a requirement for CaM in optimal Akt phosphorylation at T308 and S473 (135, 136). Thus, further investigation is warranted to clarify the functional significance of CaM binding to the AKT PH domain and to determine the precise role of this interaction in lymphocytes.

## Conclusion

The studies discussed herein highlight the essential yet complex functions of PH domain-containing proteins in lymphocytes and other immune cells. It is well established that a subset of PH domains modulate the function of their parent proteins by binding to membrane-bound lipids as well as soluble lipid analogs. Furthermore, proteins regulated in this manner, such as the PI3K effector kinases Akt and Itk, are indispensable for immune cell function. Indeed, mutations that disrupt the lipid-binding capacity of PH domains are known to result in human disease, a phenomenon perhaps best demonstrated by the immunologic defects associated with mutations in Tec family kinases. Analogous and unique pathological processes observed in animal models and *in vitro* experiments reinforce the critical role of PH domain-containing proteins in the immune system. However, evidence increasingly shows that PH domains also interact with non-lipid substrates, and these interactions can be cooperative, antagonistic, or completely independent of lipid-binding capacity. The breadth of these interactions must be elucidated in order to fully understand role of PH domain-containing proteins in immune cell function. Thus, future work should investigate the capacity of PH domains to interact with multiple substrates, including both lipids and proteins, and should include careful evaluation of how binding of each substrate affects the binding of others.

## Conflict of Interest Statement

The authors declare that the research was conducted in the absence of any commercial or financial relationships that could be construed as a potential conflict of interest.
